# A Novel Aggregation-Induced Emission-Based Electrochemiluminescence Aptamer Sensor Utilizing Red-Emissive Sulfur Quantum Dots for Rapid and Sensitive Malathion Detection

**DOI:** 10.3390/bios15010064

**Published:** 2025-01-20

**Authors:** Yajun Wu, Dongxiao Ma, Xiaoli Zhu, Fangquan Xia

**Affiliations:** Key Laboratory of Interfacial Reaction & Sensing Analysis in Universities of Shandong, School of Chemistry and Chemical Engineering, University of Jinan, Jinan 250022, China; 202221201237@stu.ujn.edu.cn (Y.W.); ma1920660278@163.com (D.M.); chm_zhuxl@ujn.edu.cn (X.Z.)

**Keywords:** sulfur quantum dots, electroluminescence, malathion, detection

## Abstract

Rapid, effective, and cost-effective methods for large-scale screening of pesticide residues in the environment and agricultural products are important for assessing potential environmental risks and safeguarding human health. Here, we constructed a novel aggregation-induced emission (AIE) electrochemical aptamer (Apt) sensor based on red-emissive sulfur quantum dots (SQDs), which aimed at the rapid screening and quantitative detection of malathion. SQDs were prepared using a two-step oxidation method with good electrochemiluminescence (ECL) optical properties. These SQDs were modified onto the electrode surface to serve as ECL luminophores. Subsequently, Apt was introduced and modified to form a double-helix structure with the complementary chain (cDNA). The ECL signal was reduced because the biomolecules had poor electrical conductivity and inefficient electron transfer. When the target malathion was added, the double helix structure was unraveled, the malathion Apt fell off the electrode surface, and the ECL signal was restored. The linear range of detection was 1.0 × 10^−13^–1.0 × 10^−8^ mol·L^−1^, and the detection limit was 0.219 fM. The successful preparation of the sensor not only develops the ECL optical properties of SQDs but also expands the application of SQDs in ECL sensing.

## 1. Introduction

Malathion, as an organophosphorus pesticide [1,2], has been widely used in pest control of rice, vegetables, and fruit trees [3,4]. However, due to the irrational use of pesticides, their residues in soil, water, and agricultural products may cause environmental pollution and food safety problems. The United States Environmental Protection Agency (EPA) stipulates a maximum residue limit (MRL) of 8 mg/kg for malathion in fruits and vegetables. In China, according to the national standards of the People’s Republic of China, the MRL for malathion in vegetables should not exceed 0.5 mg/kg, and for fruits, it should not surpass 2 mg/kg [5]. Therefore, it is very important to develop a highly sensitive and reliable method for pesticide residue detection.

Electrochemiluminescence (ECL) has been considered a powerful analytical technique for pesticide residue detection due to its chemiluminescence sensitivity and electrochemical method stability [6,7,8]. ECL strength depends on the efficiency of electron transfer between the luminant and the co-reactant [9,10]. So far, luminol [11], C_3_N_4_ [12], noble metal nanoclusters [13], tri (2,2′-bipyridine) ruthenium (II) [14], and quantum dots [15] have been used in ECL analysis as luminaries. Quantum dots are a class of materials with excellent optical properties and chemical stability. However, traditional quantum dots such as CdTe [16] and CdS quantum dots [17] rely on heavy metal elements for their luminescence, which will cause serious pollution to the environment [18]. Therefore, it is urgent to develop new quantum dots with good biocompatibility to promote the development of ECL technology. Therefore, the development of metal-free quantum dots such as carbon quantum dots, graphene quantum dots [19], and black phosphorus quantum dots [20] has attracted more and more attention. Sulfur quantum dots (SQDs) as a new type of metal-free quantum dots with good optical properties, low toxicity, and antibacterial properties, have developed rapidly in recent years and have been widely used in cell imaging, fluorescence sensing, and light-emitting diodes [21,22,23,24,25,26]. However, the luminous color of the material is only limited to the blue and green regions, and it cannot achieve red luminescence, which limits its application in optoelectronic devices and analytical detection to a certain extent.

Inspired by Tang et al., aggregation-induced luminaires have been widely developed and applied recently [27]. Aggregation-induced emission (AIE) refers to the phenomenon that a substance does not emit light or emits light weakly in the molecular or amorphous state but changes to strong emission when it is aggregated or crystallized. Due to the π-π stacking between molecules, the traditional luminous materials in the excited state return to the excited state through a non-radiative transition in the aggregated state, which greatly weakens the luminous efficiency [28]. AIE effect effectively overcomes the phenomenon of Aggregation-caused Quenching (ACQ) caused by traditional fluorophores and can solve the problem of weak luminescence and low photosensitivity. Inspired by the AIE effect, Wang’s team [29]. designed and synthesized a new sulfur quantum dot using the unique perspective of AIE and adjusted its emission color to the red direction of the spectrum. However, the research and application of red luminous SQDs is still in its infancy. Aptamer is an oligonucleotide sequence obtained through artificial selection, which exhibits high specificity and high affinity for binding to specific target molecules (such as proteins, small molecule compounds, etc.). The ECL aptamer sensor possesses advantages such as high sensitivity, strong specificity, and ease of operation [30,31,32,33,34,35,36,37,38,39].

In this study, an ECL electrochemical aptamer sensor based on red luminous SQDs was constructed for the detection of malathion. The red luminous SQDs were prepared by a two-step oxidation method. The morphologies and components of these quantum dots were characterized by FT-IR and XRD, and the ECL luminescence mechanism was speculated. The SQDs were modified on the electrode surface as ECL luminescent bodies, and then AuNPs were modified on the electrode surface to provide binding sites so that the malathion cDNA was connected to the electrode surface through the Au-NH_2_ bond, and then the modified aptamer (Apt) formed a double helix structure with cDNA. At that time, the insufficient conductivity properties of biomolecules resulted in a low electron transmission rate across the electrode, consequently the ECL signal was reduced. When the target malathion was added, the double helix structure was unraveled, so that the malathion Apt fell off the electrode surface and the ECL signal was restored. Thus, the electrochemical luminescence Apt sensor was constructed to detect malathion [40,41,42,43,44,45,46,47,48,49,50,51,52,53].

## 2. Materials and Methods

### 2.1. Chemicals and Apparatus

All pesticides used in this study were provided by the Shanghai Pesticide Research Institute Co., Ltd. (Shanghai, China). Sublimed sulfur, sodium sulfide, sodium borohydride, and ethylenediamine were purchased from Aladdin Industrial Inc. (Shanghai, China). Sodium citrate dihydrate, chloro-auric acid, potassium persulfate, sodium chloride, and potassium chloride were purchased from Sinopharm Chemical Reagent Co., Ltd. (Shanghai, China). The water used in the experiment is ultra-pure, and the reagents used are analytically pure.

The oligonucleotides used in this experiment were synthesized and purified by Shanghai Sangon Biotech Co., Ltd. (Shanghai, China). The DNA sequences used in this article were as follows: 

Malathion Apt: 5′-NH_2_-(CH_2_)_6_-ATCCGTCACACCTGCTCTTATACACAATTGTTTT TCTCTTAACTTCTTGACTGCTGGTGTTGGCTCCCGTAT-3′

Malathion cDNA: 5′-NH_2_-(CH_2_)_6_-GGGAGCCAAC ACCAG-3′

Scanning electron microscope (SEM) images using a field emission scanning electron microscope (Zeiss, Jena, Germany). Electrochemical experiments were carried out at CHI 660E Electrochemistry Station (Shanghai CH Instruments Co., Ltd., Shanghai, China). The ECL signal was measured by an MPI-B electrochemiluminescence analyzer (Xi’an Remex Analysis Instruments Co., Ltd., Xi’an, China). Freeze-drying machine (BoYiKang (BeiJing) instrument Co., Ltd., Beijing, China). Steady state/Transient fluorescence spectrometer (Edinburgh Instruments Ltd., Livingston, UK).

### 2.2. Preparation of Red Luminous SQDs

**Figure 1:**
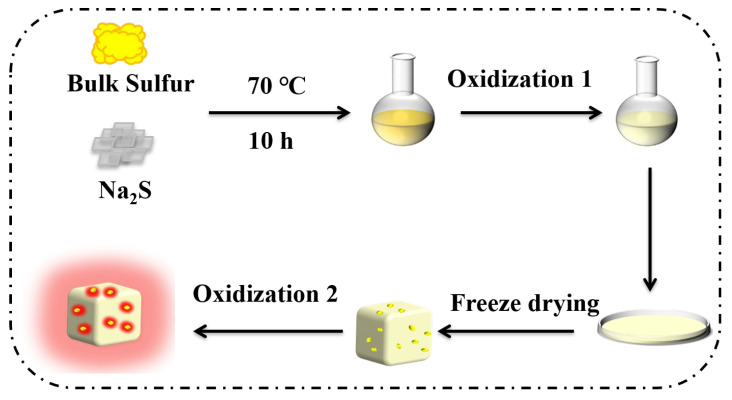
Preparation process of SQDs.

Sublimed sulfur powder (0.026 g) and Na_2_S (4.0 g) were added to ultra-pure water (15 mL) in a 50 mL round-bottomed flask and continuously stirred magnetically for 10 h under 70 °C water bath conditions, to obtain a light yellow transparent solution and complete the first step of oxidation. The solution was transferred to a surface dish and freeze-dried for 12 h to get a white powder. When the white powder was in contact with the air, the powder changed from white to brown and finally to yellow, at this time the second step oxidation process was completed, and red fluorescence was emitted under the irradiation of the ultraviolet lamp, and the preparation of SQDs was completed. After the powder was fully ground in a mortar, 30 mg of SQDs were dispersed in a dispersion solution of 5 mL water and ethanol (V water/V ethanol = 1:9) for use [29]. The preparation process of SQDs is shown in Figure 1.

### 2.3. Preparation of AuNPs

HAuCl_4_ (1%, aqueous solution, 170 μL) was added to the sodium citrate dihydrate solution (19.40 mL, 0.25 mmol·L^−1^) under the condition of magnetic stirring, and then a fresh ice NaBH4 (0.6 mL, 0.1 mol·L^−1^) solution was added, and it was left to age at 4 °C for 6 h. The light red solution of AuNPs was successfully prepared.

### 2.4. Aptamer Sensor Construction

**Figure 2:**
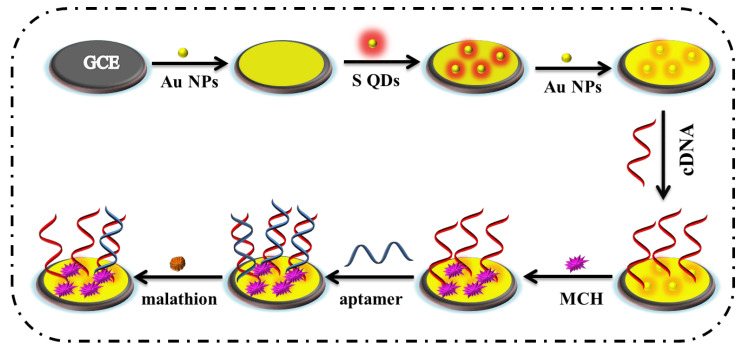
Schematic diagram of the construction of the electrochemical aptamer sensor. When malathion was added, the DNA double helix unraveled aptamer fell off, and the ECL signal recovered to realize the detection of malathion.

The treated glass carbon electrode was immersed in a gold-plating solution and gold-plated at −0.2 V (vs. Ag/AgCl) potential for 1 min using the potentiostatic *i-t* curve method to obtain AuNPs/GCE. The sulfur quantum-dot solution was evenly dispersed by ultrasound, 8 μL drops were removed and coated on the surface of the AuNPs/GCE electrode, and then dried at room temperature to obtain SQDs/AuNPs/GCE. The AuNPs/SQDs/AuNPs/GCE modified electrode was then coated with AuNPs (8 μL) on the surface of the electrode to protect the SQDs and dried at room temperature. A drop of 8 μL malathion cDNA (1 μmol·L^−1^) was dripped onto the surface of the electrode, incubated at 4 °C for 12 h, and the electrode was cleaned with PBS solution at pH 7.4. To block non-specific binding sites on the electrode, 4 μL of MCH (10 μmol·L^−1^) was added to the surface of the electrode and incubated at 37 °C for 1 hour, followed by washing with PBS solution. Lastly, 8 μL malathion Apt (1 μmol·L^−1^) was dropped onto the surface of the electrode, incubated under 37 °C for 1 h, the electrode surface was washed with PBS solution pH 7.4, Apt/MCH/cDNA/AuNPs/SQDs/AuNPs/GCE, thereby completing the preparation of the electrochemical malathion aptamer sensor. The sensor construction process is shown in Figure 2. 

### 2.5. ECL Detection Method

The surface of the constructed sensor was treated with malathion of different concentrations, incubated at 37 °C for 1 h, and then cleaned with a PBS solution with pH 7.4. In a PBS (pH 7.4) substrate containing 0.1 mol·L^−1^ of K_2_S_2_O_8_, with an aptamer sensor as a working electrode, an Ag/AgCl electrode reference electrode, and a platinum electrode auxiliary electrode, a cyclic voltammetry scan was performed in the potential range of −2.0–0.2 V. The photomultiplier had a voltage of 700 V, and the obtained light intensity signal was recorded.

## 3. Results and Discussion

### 3.1. Characterization of Red Luminous SQDs

**Figure 3:**
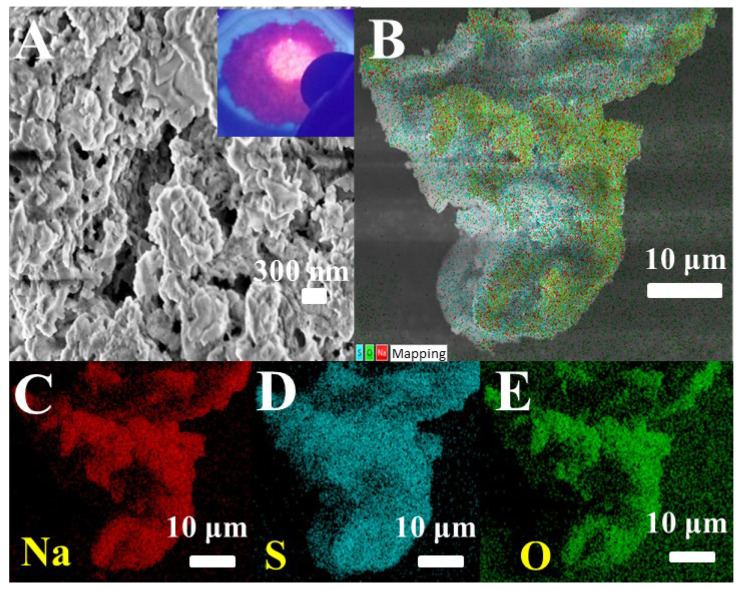
(**A**) SEM diagram of red luminous SQDs (inner illustration: SQDs luminescence diagram under UV lamp); (**B**) EDS chromatogram: Na (**C**), S (**D**), and O (**E**).

The surface morphology and components of the samples were characterized by scanning electron microscopy (SEM). Figure 3A is the SEM image of red luminous SQDs. It can be seen from the image that the morphology of red luminous SQDs is irregular layered material. The sample emits bright red light when irradiated by the ultraviolet lamp (illustration in Figure 3A). The EDS spectrum in Figure 3B–E shows the composition and distribution of SQDs elements. The main elements were Na, O, and S, which is consistent with previous reports [29], indicating the successful preparation of SQDs.

**Figure 4:**
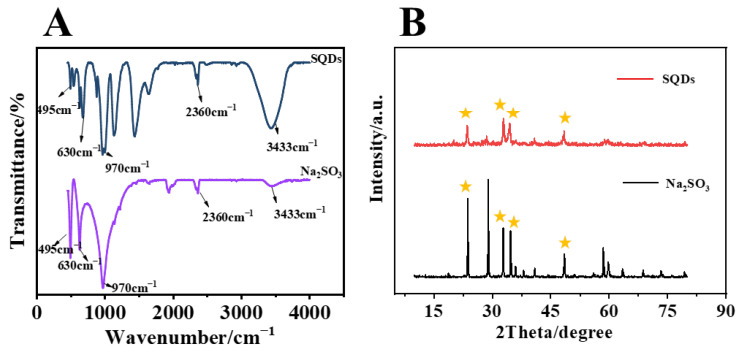
(**A**) Infrared spectra of SQDs and Na_2_SO_3_. (**B**) Comparison of XRD spectra of SQDs and Na_2_SO_3_. The infrared characteristic peaks are similar, and the XRD diffraction peaks correspond to the same. Yellow stars are marked as characteristic peak positions.

To verify the composition and structure of SQDs, the SQDs were characterized by an infrared absorption spectrum and XRD. Figure 4A shows the infrared spectra of SQDs and Na_2_SO_3_. It can be seen from the Figure that the SQDs had characteristic peaks similar to those of Na_2_SO_3_ at 495, 630, 970, 2360, and 3433 cm^−1^, respectively. In addition, the 4B XRD comparison between SQDs and Na_2_SO_3_ is shown in Figure 4B, where SQDs have sharp diffraction peaks at 23.6°, 32.8°, 34.5°, and 48.4°, corresponding to the characteristic peaks of Na_2_SO_3_. Therefore, it can be preliminarily speculated that the main component of red luminous SQDs was Na_2_SO_3_.

**Figure 5:**
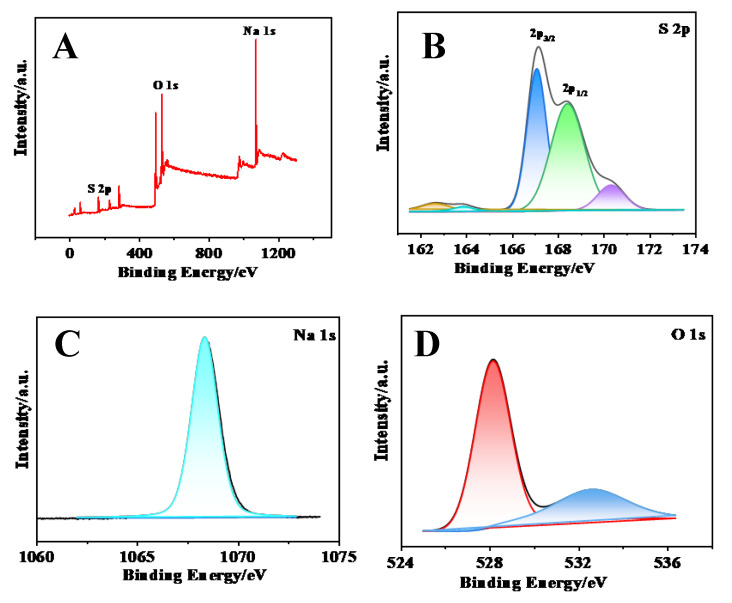
(**A**) XPS total spectrum and XPS analysis of (**B**) S2p, (**C**) Na1s, and (**D**) O1s regions. The structure of SQD is elemental sulfur wrapped in sodium sulfite.

The composition of SQDs was further analyzed by XPS spectroscopy. Figure 5A shows the total XPS spectrum of SQDs, which contain peaks of S2p, Na1s, and O1s. The two main peaks of S2p are located at 167 eV (Figure 5B blue peak) and 168.4 eV (Figure 5B green peak), representing S2p_3/2_ and S2p_1/2_ of SO_3_^2−^, the peak of binding energy at 170.3 eV (Figure 5B purple peak) indicates the surface oxidation of sulfites to SO_3_^−^, and the peak of 161.4–164.7 eV indicates the presence of elemental sulfur (Figure 5B). The peak of Na1s appears at 1071.3 eV (Figure 5C blue peak). The peaks at 531.1 eV (red peak) and 535.7 eV (blue peak) in the O1s map can be attributed to the peaks of the -OH and S=O groups, respectively (Figure 5D). It is inferred that the structure of SQDs was the elemental sulfur wrapped in sodium sulfite.

### 3.2. Discussion on ECL Luminescence Mechanism

**Figure 6:**
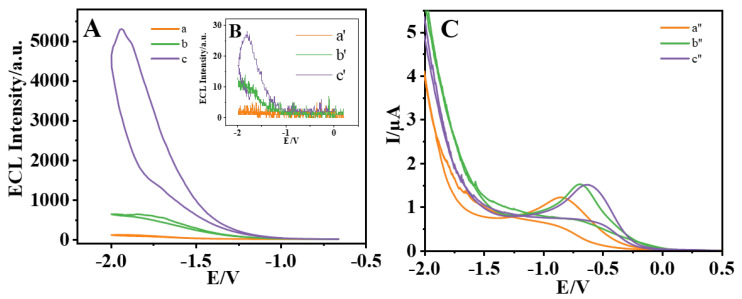
The ECL potential of different modified ECL intensity (**A**,**B**) and cyclic voltammograms (**C**) GCE (a, a′, a″), Au NPs/GCE (b, b′, b″), and MCH/cDNA/AuNPs/SQDs/AuNPs/GCE (c, c′, c″) in PBS solutions containing 0.1 mol·L^−1^ K_2_S_2_O_8_ (**A**,**C**) and without 0.1 mol·L^−1^ K_2_S_2_O_8_ (**B**).

By studying the GCE, AuNPs/GCE, and MCH/cDNA/AuNPs/SQDs/AuNPs/GCE electrodes in including and excluding K_2_S_2_O_8_ pH 7.4 0.1 mol·L^−1^, the ECL mechanism of SQDs/K_2_S_2_O_8_ system was investigated. The experimental results are shown in Figure 6. In the PBS solution without K_2_S_2_O_8_ (pH 7.4), GCE had no obvious luminous signal (Figure 6B, curve a′). When AuNPs were modified onto the bare electrode by electrochemical deposition, due to their good conductivity and catalytic properties, AuNPs could produce weak luminescence in the PBS solution, about 13 a.u. left and right (Figure 6B, curve b′). When the SQDs were modified to the electrode surface, the ECL intensity was about 30 a.u. and the luminescence intensity was further enhanced (Figure 6B, curve c′). In the PBS solution containing K_2_S_2_O_8_ (pH 7.4), naked GCE exhibits an ECL intensity of about 116 a.u (Figure 6A, curve a), which is inferred to be 1(O_2_)_2_* [54] emission in the O_2_/S_2_O_8_^2−^ system. When AuNPs were modified onto the bare electrode, the reduction peak of the CV curve shifted positively by about 0.2 V (Figure 6C, curve b″) due to the increase in the charge transfer rate caused by the introduction of AuNPs on the bare electrode, and the luminous intensity increased to about 846 a. u compared with that of the bare electrode (Figure 6A, curve b). When SQDs were modified on the electrode surface, the ECL intensity increased sharply to about 5400 a.u. (Figure 6A, curve c). This was because S_2_O_8_^2−^ as a co-reactive substance can enhance the luminescence performance of SQDs, the ECL luminescence mechanism of SQDs/K_2_S_2_O_8_ system is as follows:

SQDs + e**^−^ →** SQDs^•−^ (1)

S_2_O_8_^2−^ + e**^−^ →** SO_4_^2−^ + SO_4_^•−^ (2)

SQDs^•−^ + SO_4_^•**−**^
**→** SQDs* + SO_4_^2−^ (3)

SQDs* **→** SQDs + *hυ* (4)

### 3.3. AC Impedance Characterization of Aptamer Sensor

**Figure 7:**
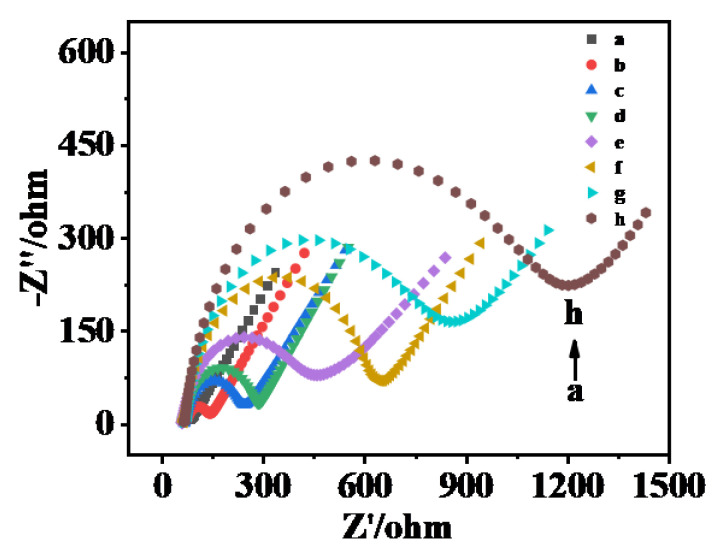
AC impedance spectra of different modified electrodes. a–h: AuNPs/GCE, GCE, AuNPs/SQDs/AuNPs/GCE, SQDs/AuNPs/GCE, cDNA/AuNPs/SQDs/AuNPs/GCE, MCH/cDNA/AuNPs/SQDs/AuNPs/GCE, malathion/Apt/MCH/cDNA/AuNPs/SQDs/AuNPs/GCE, Apt/MCH/cDNA/AuNPs/SQDs/AuNPs/GCE. AC impedance was measured in a solution containing 0.1 mol·L^−1^ perchlorate and 5.0 mol·L^−1^ [Fe (CN) 6]^3−/4−^.

In a [Fe(CN)_6_]^3−/4−^ solution containing 0.1 mol·L^−1^ KCl and 5.0 mmol·L^−1^, the successful assembly of the biosensor layer on the electrode surface was verified using electrochemical impedance spectroscopy (EIS) (illustrated as equivalent circuit diagram). As can be seen from Figure 7, the impedance of GCE (curve b) was 110.4 Ω, and when AuNPs (curve a) were modified on the electrode surface, the impedance value decreased to 30.06 Ω due to its high electrical conductivity. SQDs (curve c) were further modified on the electrode surface, and the impedance value increased to 311 Ω due to the poor conductivity of the quantum dot. When AuNPs (curve d) were modified twice on the electrode surface, the electrode impedance was reduced to 158 Ω. When cDNA (curve e), MCH (curve f), and Malathion Apt (curve h) were modified layer upon layer on the surface of AuNPs/SQDs/AuNPs/GCE electrodes, the electron transport rate on the surface of the electrodes was hindered due to the non-conductivity of the biological material itself and the increase in the number of modified layers, and the impedance values successively became 402.6 Ω, 550.5 Ω, and 868.3 Ω. When malathion was dropped onto the electrode (curve g), the Apt specifically bound to malathion and fell off the electrode, reducing the electron transfer resistance and decreasing the impedance value to 664 Ω. The above results show that the layer modification of the sensor was successful.

### 3.4. Optimization of Experimental Conditions

To explore the optimal conditions for detecting malathion by an aptamer sensor, the effects of different synthesis times, pH of the substrate, potassium persulfate concentration, and incubation time of SQDs on ECL intensity were investigated. To improve the sensitivity of the aptamer sensor, a PBS solution containing K_2_S_2_O_8_ was used as the base solution, and 10^−9^ mol·L^−1^ of pesticide was added to the surface of the electrode for detection.

#### 3.4.1. Synthesis Time of Quantum Dots

**Figure 8:**
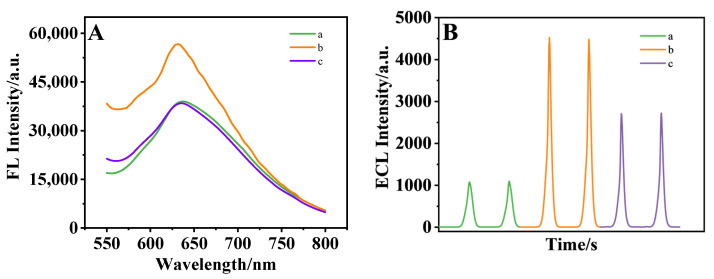
(**A**) Fluorescence spectra and (**B**) ECL intensity of SQDs at different synthesis times a, b, c: 3 h, 10 h, and 48 h. The ECL intensity indicates that the optimal synthesis time of SQDs was 10 h.

The fluorescence of red luminous SQDs at different synthesis times was characterized by a transient/steady-state fluorescence spectrometer, and the absolute quantum yield was calculated. As shown in Figure 8A, curves a, b, and c are fluorescence spectra of red luminous SQDs synthesized by 3 h, 10 h, and 48 h heating, respectively. It can be seen from the Figure that under the excitation wavelength of 350 nm, the emission wavelength of the quantum dot is about 635 nm, which is a red wavelength. The fluorescence intensity of SQDs is the highest at the synthesis time of 10 h. In addition, the absolute fluorescence quantum yields of 3 h, 10 h, and 48 h red luminous SQDs are 2%, 7%, and 3%, respectively. The synthesis time of SQDs affects the ECL performance of the sensor. It can be seen from Figure 8B that the ECL intensity reached its maximum at the synthesis time of 10 h. Therefore, the optimal synthesis time of SQDs was 10 h.

#### 3.4.2. pH of the Bottom Solution

**Figure 9:**
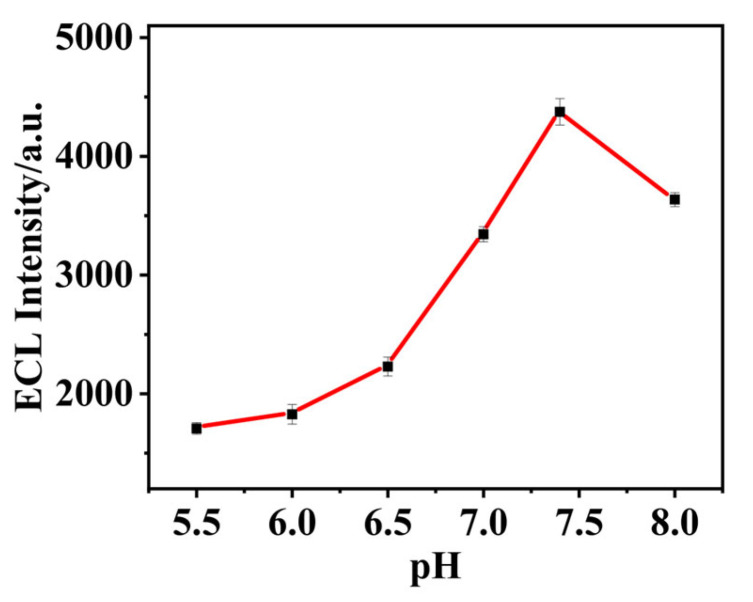
Effect of substrate pH on the performance of the aptamer sensor. The optimal pH value of the substrate was 7.4.

pH is an important factor affecting the performance of aptamer sensors because it affects the activity of biological materials as well as the structure and properties of the Apt. With other conditions unchanged, the pH of the bottom solution was adjusted with 0.1 mol·L^−1^ HCl and 0.1 mol·L^−1^ NaOH solution, and the ECL intensity of the bottom solution was measured at pH values of 5.8, 6.2, 6.6, 7.0, 7.4, and 7.8. The experimental results are shown in Figure 9. When the pH value of the substrate increased in the range of 5.5–7.4, the ECL signal increased. When the pH value was higher than 7.4, the ECL signal decreased with increasing pH. Therefore, the optimal pH value of the substrate was 7.4.

#### 3.4.3. Potassium Persulfate Concentration

**Figure 10:**
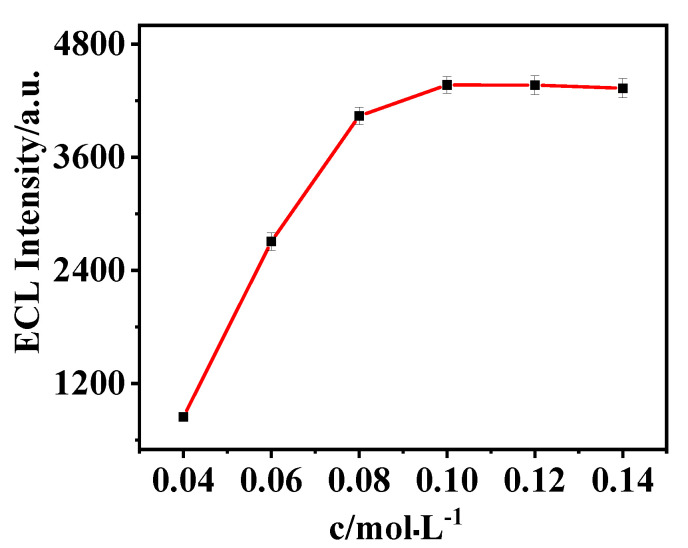
Influence of substrate K_2_S_2_O_8_ concentration on the performance of the aptamer sensor. The optimal concentration of K_2_S_2_O_8_ was 0.10 mol·L^−1^.

The concentration of potassium persulfate was 0.04, 0.06, 0.08, 0.10, 0.12, and 0.14 mol·L^−1,^ and the influence of potassium persulfate concentration on ECL intensity was studied. The experimental results are shown in Figure 10. ECL intensity increased with the increase of potassium persulfate concentration, and ECL intensity reached the maximum when the concentration of K_2_S_2_O_8_ increased to 0.10 mol·L^−1^. When the concentration of K_2_S_2_O_8_ exceeded 0.10 mol·L^−1^, the ECL signal intensity tended to stabilize. Therefore, the optimal concentration of K_2_S_2_O_8_ was 0.10 mol·L^−1^.

#### 3.4.4. Incubation Time of Apt and cDNA

**Figure 11:**
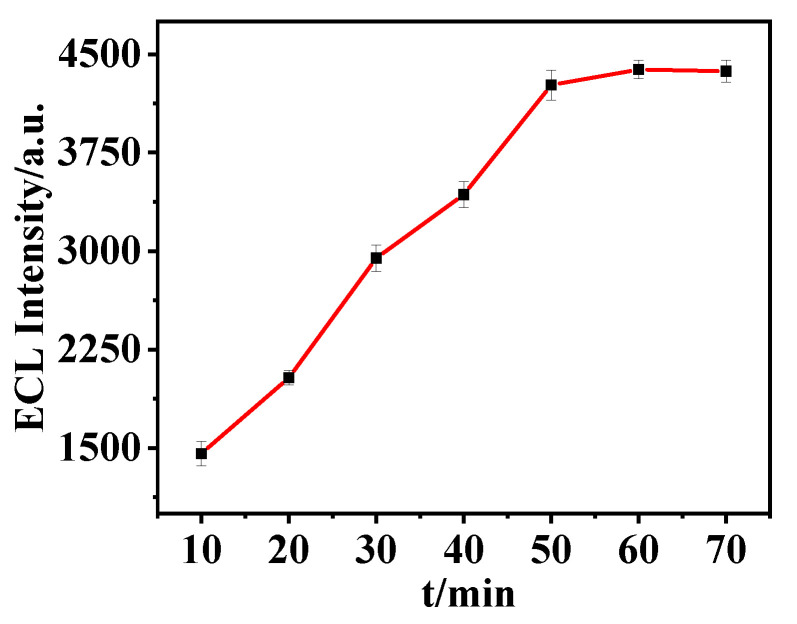
Effect of incubation time on the performance of the aptamer sensor. The optimal incubation time was 60 min.

Incubation time affects the hybridization efficiency between Apt and cDNA. To obtain the best detection effect of the aptamer sensor, the incubation time of Apt and cDNA was studied. Under the premise of keeping other conditions unchanged, the incubation time of the control the Apt and the cDNA was 10, 20, 30, 40, 50, 60, and 70 min, and the ECL test was performed. The experimental results are shown in Figure 11. With the increase of incubation time, ECL intensity gradually increased and reached the maximum value at 60 min. Therefore, the optimal incubation time was 60 min.

### 3.5. Analysis Performance of the Aptamer Sensor

**Figure 12:**
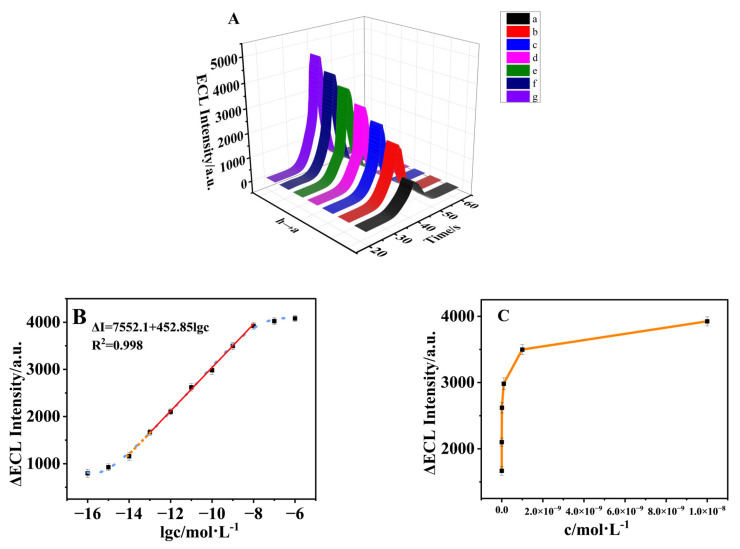
(**A**) ECL intensity of malathion with different concentrations. a–g: 0, 10^−13^, 10^−12^, 10^−11^, 10^−10^, 10^−9^, and 10^−8^ mol·L^−1^, The curve did not show a linear relationship in the detection of higher (10^−16^, 10^−15^ mol·L^−1^) and lower concentrations (10^−7^, 10^−6^ mol·L^−1^) of malathion (blue curve). (**B**) The calibration plot of the ECL sensor. (**C**) ECL intensity–concentration plot.

Under the optimal detection conditions, different concentrations of malathion were modified on the surface of the sensor to verify the analytical performance of the sensor. As can be seen from Figure 12A, the electroluminescence intensity of the aptamer sensor without malathion pesticide was very low, about 1000 a.u. (Curve a). When malathion pesticide was dropped onto the electrode, the ECL intensity gradually increased with the increase of malathion concentration. This was because with the increase of malathion concentration, more and more malathion specifically bound to the Apt, the double-helix structure was unraveled, the Apt fell off from the electrode surface, and the electron mass transfer rate was accelerated. Therefore, the ECL intensity was positively correlated with the concentration of malathion. As illustrated in Figure 12B, the linear equation is ∆*I* = 7552.1 + 452.85*lgc*, R^2^ = 0.998, the linear range of detection is 1.0 × 10^−13^–1.0 × 10^−8^ mol·L^−1^, the limit of detection (LOD) is 0.219 fM (S/N = 3), and the limit of quantitation (LOQ) is 0.0398pM (S/N = 10). When the concentration was 10^−14^ mol·L^−1^, although the linear relationship was also followed, there were data with large deviations, resulting in large relative errors in the signal. The curve tended to flatten out and did not show a linear relationship in the detection of higher (10^−16^, 10^−15^ mol·L^−1^) and lower concentrations (10^−7^, 10^−6^ mol·L^−1^) of malathion.

### 3.6. Selectivity, Stability, and Reproducibility of Aptamer Sensors

**Figure 13:**
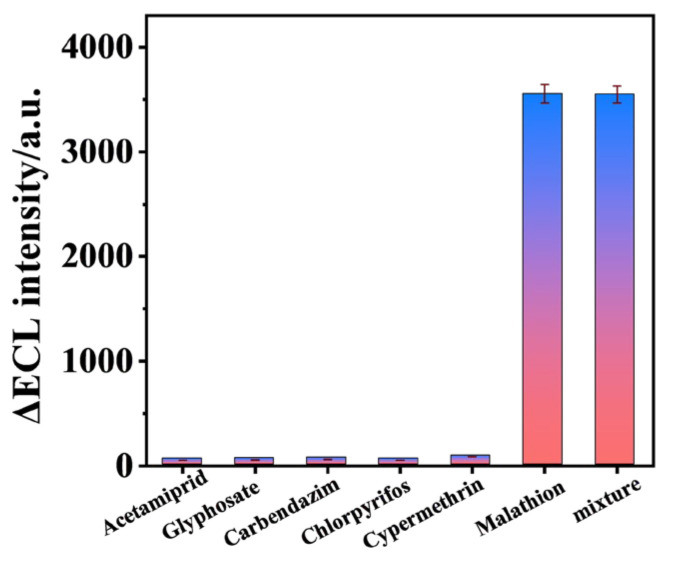
Selectivity of the aptamer sensor. The sensor had good selectivity for malathion.

To test the selectivity of the aptamer sensor, different pesticides of 10^−6^ mol·L^−1^ (including acetamiprid, glyphosate, carbendazim, chlorpyrifos, cypermethrin, and a combination of pesticides containing malathion) and malathion (10^−9^ mol·L^−1^) were applied to the electrode surface for incubation, and the ∆ECL intensity before and after pesticide addition was measured. As shown in Figure 13, electrodes dripping with other pesticides produced very low ∆ECL intensity. ECL intensity was obviously enhanced when the malathion pesticide was dropped onto the electrode surface. When the mixed pesticide was added to the sensor surface, ECL intensity was almost the same as that when the malathion pesticide was dropped alone, indicating that the sensor had good selectivity.

**Figure 14:**
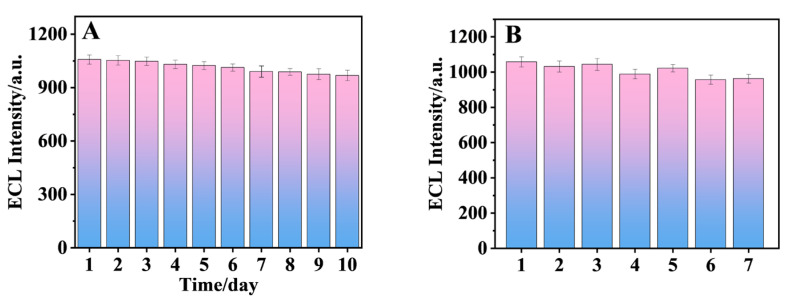
(**A**) Stability and (**B**) reproducibility of the aptamer sensor. The sensor could stabilize the signal response for 10 days and had good repeatability.

The sensor was exposed to 4 °C for 10 days, and the ECL response was measured daily to check the stability of the sensor. As shown in Figure 14A, the ECL intensity of the electrode after 10 days was 91.5% of the original ECL intensity, indicating that the sensor had excellent stability. Figure 14B shows the results of parallel experiments with seven sensors, and the relative standard deviation of peak intensity was 3.95%. The results show that the sensor had good reproducibility.

### 3.7. Real Sample Analysis

Samples of vegetables and fruits purchased from local supermarkets were tested for actual sample analysis. Firstly, oranges, cabbages, and eggplants were accurately weighed to 100 g each, and ground into juice using a juicer. The juice samples were then dispersed in 100 mL of 0.1 mol·L^−1^ PBS (pH 7.4), centrifuged at 8000 rpm for 5 min, and the resulting supernatant was obtained for detection. Initially, malathion was not detected in the sample solutions by HPLC-MS. Subsequently, a quantitative standard of malathion was added to the samples, which were then processed according to the aforementioned method. By spiking different samples with known concentrations of malathion for recovery tests, the accuracy of the analytical method was verified. As can be seen from Table 1, the recovery rate of malathion ranged from 95% to 104%, and the relative standard deviation was less than 6%. The results show that the sensor had good accuracy, stability, and repeatability, and could effectively be used for the detection of malathion in real samples.

## 4. Conclusions

In this chapter, red-emitting SQDs were synthesized by a two-step oxidation method, and the morphology, composition, and structure of the quantum dots were characterized by SEM, EDS, FT-IR, XRD, transient-steady-state fluorescence, and XPS, which successfully verified that the quantum dots were the main structure of the Na_2_SO_3_ protective matrix containing elemental sulfur. In addition, red luminous SQDs also had good ECL optical properties. Under the optimal experimental conditions, an ECL aptamer sensor based on SQDs was successfully developed, and malathion was successfully detected by using the principle that the steric hindrance effect would limit electron transfer and reactant diffusion on the electrode surface. The preparation of the sensor not only develops the ECL optical properties of SQDs but also expands the application of SQDs in ECL sensing.

## Figures and Tables

**Table 1:** Determination of malathion in various samples.

Samples		Added (n·g^−1^)	HPLC-MS (ng·g^−1^)	ECL (n = 3)		
				Found (ng·g^−1^)	Recovery (%)	RSD (%)
Orange	1^#^	0.0	Not found	--	--	--
	2^#^	1.00	0.98	1.02	102	6
	3^#^	5.00	4.90	4.98	99.4	5
Cabbage	1^#^	0.0	Not found	--	--	--
	2^#^	1.00	1.01	0.95	95	3
	3^#^	5.00	5.10	5.20	104	4
Eggplant	1^#^	0.0	Not found	--	--	--
	2^#^	1.00	0.98	0.97	97	4
	3^#^	5.00	4.60	4.80	98.0	5

## Data Availability

The data are contained within the article.
